# Right ventricular strain in patients with tetralogy of Fallot

**DOI:** 10.1186/1532-429X-13-S1-P204

**Published:** 2011-02-02

**Authors:** Michael D Taylor, Kan N Hor, Wojceich Mazur, D Woodrow Benson, William M Gottliebson

**Affiliations:** 1 Cincinnati Children's Hospital Medical Center, Cincinnati, OH, USA; 2 Ohio Heart and Vascular Center, Cincinnati, OH, USA

## Objective

The primary objective was to measure the right ventricular longitudinal strain in a cohort of repaired TOF patients with pulmonary regurgitation using a recently validated feature tracking algorithm.

## Background

Left ventricular (LV) circumferential strain shows cardiac dysfunction earlier than ejection fraction. However, measuring right ventricle strain is technically challenging due to the thin RV wall and complex morphology. Also, the predominant RV strain is longitudinal, whereas the predominant LV strain is circumferential. We recently validated a feature tracking algorithm for evaluation of left ventricular strain. Based on that work, we hypothesized that feature tracking would provide accurate and reproducible RV strain metrics for use in longitudinal evaluation of repaired TOF patients.

## Methods

Forty patients and sixteen normal controls were evaluated. All patients had pulmonary regurgitant fraction > 30% with no residual obstruction. We dichotomized the TOF patients by RVEF (< /= or > 55%) for analysis. CMR image analysis included routine volumetry. Circumferential LV and longitudinal RV strain were calculated using the previously validated feature tracking algorithm. The average strain at the mid-ventricular level was used for comparison. Means were compared using student's t-test and variances were compared with the Lev1:med test.

## Results

**Figure 1:**
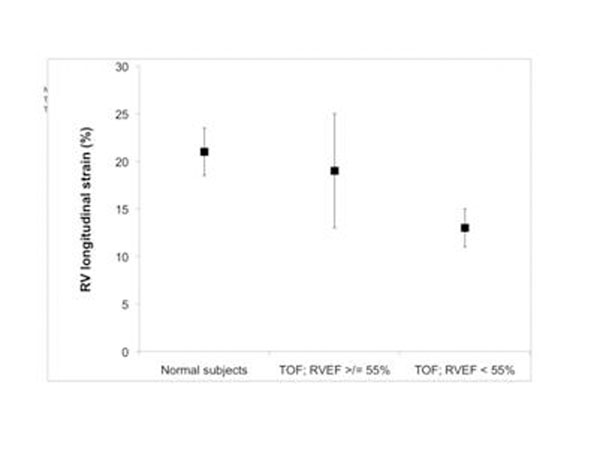


All images yielded interpretable LV circumferential and RV longitudinal strain curves. All control subjects had normal biventricular ejection fraction. They had mean longitudinal right ventricular strain = -21+/-5%. The mean RV strain of patients with TOF and normal EV was -19 +/- 12%, not statistically different than normal. However, the variance in the strain as group was 2.5x higher than controls (p<0.001). Also, there was marked dispersion for the regional strain within individual patients. The patients with abnormal RVEF had significantly lower LV circumferential and RV longitudinal than control subjects or TOF patients with normal function. There was a good correlation between strain and EF for abnormal EF (R=0.91, p=0.02), but not for patients with normal EF (R=0.22, p=0.3). Figure 1

## Conclusions

CMR feature tracking is a validated method for LV strain assessment that can be used reliably for the RV. TOF patients with normal right ventricular function (EF > 55%) have markedly variable strain with increased dispersion in regional strain values. TOF patients with abnormal right ventricular function (EF< 55%) have significantly decreased longitudinal strain and increased strain dispersion. Feature tracking with CMR for strain measurements will be used in a longitudinal evaluation of TOF patients.

